# Long-Term Outcomes of Childhood Acute Lymphocytic Leukemia Treated with Adapted Berlin–Frankfurt–Münster (BFM) Protocols: A Multicentric Analysis from a Developing Country

**DOI:** 10.3390/cancers16162898

**Published:** 2024-08-21

**Authors:** Patricia Regina Cavalcanti Barbosa Horn, Marilza de Moura Ribeiro-Carvalho, Alice Maria Boulhosa de Azevedo, Adriana Martins de Sousa, Simone Faria, Cristina Wiggers, Soraia Rouxinol, Marcia Trindade Schramm, Bárbara Sarni Sanches, Nathalia Lopez Duarte, Teresa de Souza Fernandez Seixas, Bernadete Evangelho Gomes, Elen de Oliveira, Leonardo Javier Arcuri, Elaine Sobral da Costa, Marcelo Gerardin Poirot Land, Maria Helena Faria Ornellas de Souza

**Affiliations:** 1 Department of Hematology, Universidade Estadual do Rio de Janeiro, Rio de Janeiro 20559-900, Brazil; marilza.carvalho@medicina.uerj.br (M.d.M.R.-C.); simonedefariamaia@yahoo.com.br (S.F.); tinawigg24@yahoo.com.br (C.W.); mariahelenaornellas@gmail.com (M.H.F.O.d.S.); 2 Bone Marrow Transplantation Unit, Instituto Nacional de Cancer, Rio de Janeiro 20230-130, Brazil; teresafernandez@inca.gov.br (T.d.S.F.S.); bgomes@inca.gov.br (B.E.G.); leonardojavier@gmail.com (L.J.A.); 3 Pediatric Hematolgy Department, Universidade Federal do Rio de Janeiro, Rio de Janeiro 21941-617, Brazil; alicemazevedo@gmail.com (A.M.B.d.A.); adrisousa74@gmail.com (A.M.d.S.); barbarasarni@hotmail.com (B.S.S.); nathalialopezduarte@gmail.com (N.L.D.); elenoliveira@ippmg.ufrj.br (E.d.O.); elainesc.ufrj@gmail.com (E.S.d.C.); land.marcelo@gmail.com (M.G.P.L.); 4 Pediatric Hematology Department, Hospital Federal da Lagoa, Rio de Janeiro 22470-050, Brazil; sorouxinol@gmail.com; 5 Hematology Department, Instituto Nacional de Cancer, Rio de Janeiro 20230-130, Brazil; marciaschramm@hotmail.com; 6 National Institute of Science and Technology in Childhood Cancer Biology and Pediatric Oncology, Universidade Federal do Rio Grande do Sul, Porto Alegre 90010-150, Brazil; 7 Hospital Israelita Albert Einstein, Academic Research Organization, São Paulo 01305-000, Brazil

**Keywords:** acute lymphoblastic leukemia, pediatric hematology/oncology, developing countries

## Abstract

**Simple Summary:**

Berlin–Frankfurt–Münster (BFM) protocols are widely used outside clinical trials to treat pediatric acute lymphoblastic leukemia patients. However, local specificities might demand treatment adaptations, like the reduction of high-dose methotrexate due to lack of pharmacokinetic monitoring, the substitution of conventional asparaginase for peg-asparaginase because of the unavailability of the latter, or flow-cytometry-based measurable residual disease instead of a PCR-based one. Here, we report the results of a 22-year period of children treated with BFM protocols in a developing country. The results were somewhat comparable to the BFM reports, and we conclude that BFM protocol adaptations can be safely implemented in developing countries, accounting for local specificities.

**Abstract:**

Introduction: The objective of the current study was to determine the survival probabilities of children and adolescents with acute lymphocytic leukemia treated with adapted Berlin–Frankfurt–Münster (BFM) protocols and compare our results with the original BFM reports. Methods: This retrospective study included 695 patients up to 19 years old treated with adapted BFM protocols between 1997 and 2018 in four hospitals in Rio de Janeiro. The 1997–2007 and 2008–2018 cohorts were analyzed separately. Results: More than half of the patients were stratified into the high-risk BFM classification. Overall and event-free survivals were, in the 1997–2007 period, respectively, 88% and 80% (BFM standard risk group—SRG), 75% and 67% (intermediate risk group—IRG), and 48% and 33% (high-risk group—HRG). The corresponding numbers for the 2008–2018 period were 93% and 84% (SRG), 75% and 63% (IRG), and 64% and 57% (HRG). In the second period, both the OS (HR = 0.71, *p* = 0.011) and EFS (HR = 0.62, *p* < 0.001) were higher. Except for the intermediate-risk group, the latter results are comparable to the BFM. Conclusion: The BFM protocol adaptations can be safely implemented in developing countries, accounting for local specificities.

## 1. Introduction

Acute lymphoblastic leukemia (ALL) is characterized by genetic modifications that impair differentiation and promote the proliferation of lymphoid precursor cells, which is important for risk stratification [1]. Although ALL incidence is lower in adolescents and young adults (AYA) compared with children, survival and long-term prognosis are poorer for AYA [2]. Likewise, and despite novel drugs and newer protocols, age still has an important prognostic value, and children older than 10 years fare poorer compared with younger ones. Current pediatric ALL protocols achieve a 5-year overall survival (OS) of up to 90%.

The incidence of ALL varies depending on the country, and it seems to be higher among Hispanics [3]. In Costa Rica, for example, the incidence of ALL was 56 per million children younger than 15 years, and Brazil, in 2022, reported an incidence of 53 per million in young adults [3,4]. Moreover, Mexico, California, and Florida have the highest ALL incidence rates in North America [5].

BFM protocols have long been used in Brazil. Dismal results [6] have been reported in adolescents and young adults (AYA). Poor results have also been achieved in children in Brazil with BFM protocols [7].

In the 1980s, pediatric hospitals in Rio de Janeiro, Brazil, adopted BFM-based protocols seeking a more effective treatment since, by that time, survival was very poor. Four hospitals in Rio de Janeiro have been trying to unify the BFM-adapted protocols to improve survival and to build a local ALL network. Here, we report a 22-year analysis of a large cohort of children with ALL treated at these four institutions. Our aim was to estimate the survival probabilities and characterize the main prognostic factors for children and adolescents with ALL in Rio de Janeiro, as well as indirectly compare those results with other BFM studies. This is the first multicentric survival analysis study for ALL in children at Rio de Janeiro, and we report the results of this collaboration here.

## 2. Methods

This is a multicentric retrospective study of children and adolescents younger than 19 years who began treatment with BFM-based protocols between 1997 and 2018 in any of the following four institutions in Rio de Janeiro, Brazil: Hospital Federal da Lagoa (HFL), Hospital Universitário Pedro Ernesto (HUPE/UERJ), Instituto Nacional de Cancer (INCA/MS), and Hospital de Pediatria e Puericultura Martagão Gesteira (IPPMG/UFRJ). Patients treated with protocols other than BFM-based ones were excluded. All but one institution treated all patients with BFM-based protocols.

Patients were stratified by period, namely from January 1997 to December 2007 (1997–2007) or from January 2008 to December 2018 (2008–2018). The treatment protocols were based on BFM-AEIOP 90, 95, 02, 09, and 13. The main adaptations included (a) lower methotrexate dose, 2 g/m^2^ instead of 5 g/m^2^, because of unavailability of methotrexate in vivo monitoring, (b) substituting conventional asparaginase for peg-asparaginase, which has been only recently approved in Brazil, and (c) flow-cytometry-based measurable residual disease (MRD), which was not centrally reviewed, since PCR-based MRD was seldom available in these institutions. The risk definition was based on the 8th (D8), 15th (D15), and 33rd day (D33), and 12th week (12-week) assessments, according to each original BFM protocol, and the patients were stratified into standard-risk (SRG), intermediate-risk (IRG), and high-risk (HRG) groups. The criteria for each risk group are slightly different from one protocol to the following one, but it is usually based on molecular analysis, cytogenetics, prednisone response, MRD at D15 (≥10% defines poor MRD response), and complete response (<5% blasts on D33). Prednisone good response (PGR) is defined as <1 × 10^9^/L peripheral blasts at D8 and prednisone poor response (PPR) otherwise.

The study was conducted in accordance with the Declaration of Helsinki, and the protocol was approved by all local Ethics Committees, which waived informed consent.

### Statistical Analysis

Continuous variables were reported as mean and standard deviation or median and interquartile range and range and compared with a Student’s *t* test or Mann–Whitney, as appropriate. We reported the frequency and percentages for the categorical variables and compared them with Pearson’s chi-square or Fisher’s exact test. Overall (OS) and event-free survival (EFS) curves were built with the Kaplan–Meier method and compared with the logrank test. OS was defined from diagnosis to death or last follow-up. EFS was defined as death, relapse, or secondary neoplasm, whichever occurred first. Univariable analyses were also performed with Cox models. The Cox proportional hazard assumption was checked. Multivariable Cox models were built based on the lowest Akaike information criteria (AIC). Multicollinearity was checked based on the variance inflation factor or whether the variable was already included in another collected scoring system. Cranial radiotherapy, which is a time-dependent variable, was analyzed separately using landmark analysis (in which only patients scheduled for the variable were included). All analyses were performed with R (R Foundation for Statistical Computing, Vienna, Austria), version 4.3.2.

## 3. Results

Table 1 describes the patients’ characteristics. In brief, there was a slight male predominance, and most patients were between 1 and 9 years old. As expected, B-ALL was by far the most frequent immunophenotype. More than half of the patients in both periods were classified as high risk according to the BFM risk stratification. The 285 patients treated between 1997 and 2007 were followed for a median of 11.6 years, while the 410 patients included from the 2008–2018 period were followed for a median of 4.2 years.

### 3.1. First Period (1997–2007)

**Figure 1:**
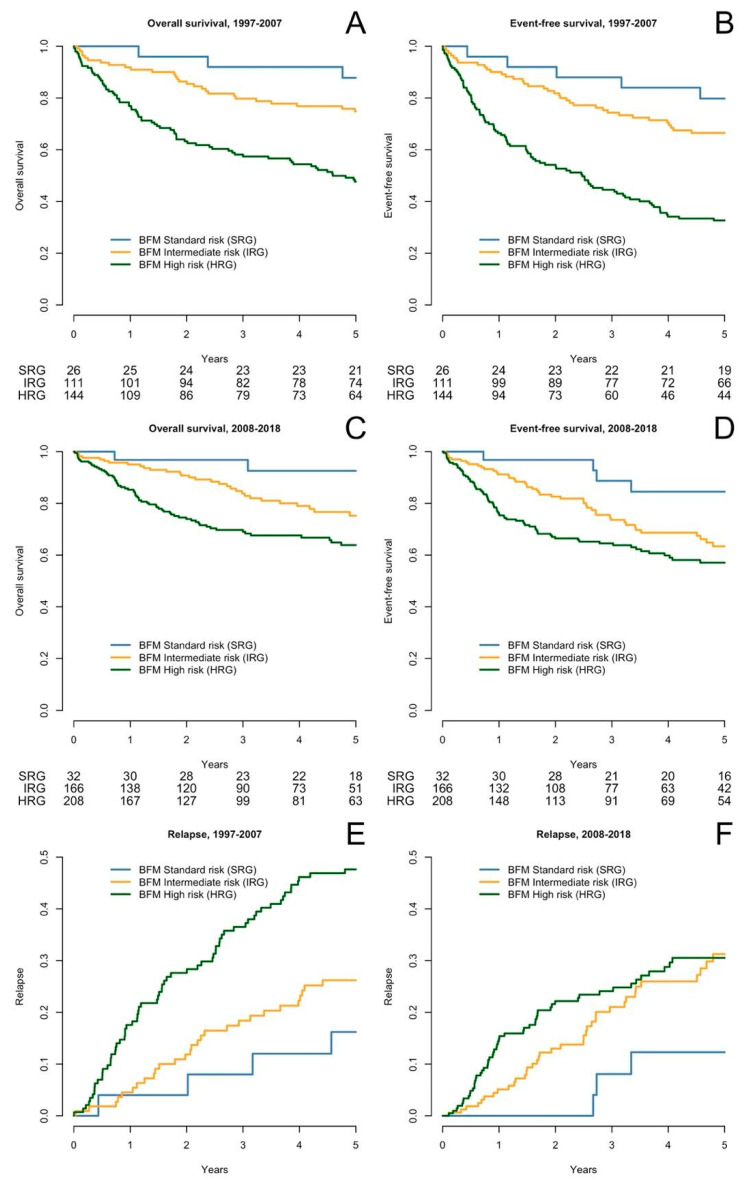
Overall and event-free survival and relapse rate according to period. OS (**A**), EFS (**C**), and relapse (**E**) in the 1997–2007 period; OS (**B**), EFS (**D**), and relapse (**F**) in the 2008–2018 period. In the relapse rate figures, the Y axis ranges from 0 to 50%.

A total of 285 patients were included. The patients’ characteristics are in Table 1. The five-year OS was 62% (95CI 56–68%). The five-year EFS was 50% (95CI 44–56%). OS, EFS, and relapse, according to BFM risk stratification, are depicted in Figure 1.

In OS univariable analyses (detailed in Table 2), male sex; age < 1 y/o or ≥10 y/o; white blood counts (WBC) > 10 × 10^9^/L, compared with ≤10 × 10^9^/L; BFM high-risk group, compared with standard risk group; NCI high-risk groups; intermediate and poor cytogenetic risk, compared with good risk; and prednisone poor response was significantly associated with poorer OS. In the multivariable analysis (Table 3), only male sex (HR = 1.60, *p* = 0.015, compared with females), age < 1 y/o or ≥10 y/o (HR = 1.56, *p* = 0.021, compared with 1–9 y/o), BFM high-risk group (HR = 4.77, *p* = 0.0025, compared with standard risk group) independently predicted poorer OS.

The variables significantly associated with poorer EFS univariable analyses (Table 4) were male sex; age < 1 y/o or ≥10 y/o; CNS+; white blood counts (WBC) > 50 × 10^9^/L, compared with ≤10 × 10^9^/L; BFM high-risk group, compared with standard risk; NCI high-risk groups; poor cytogenetic risk, compared with good risk; and prednisone poor response. In the multivariable analysis, male sex (HR = 1.79, *p* = 0.012, compared with females), age < 1 y/o or ≥10 y/o (HR = 1.57, *p* = 0.033, compared with 1–9 y/o), and BFM high-risk group (HR = 3.38, *p* = 0.042, compared with standard risk group) were significantly associated with poorer EFS.

### 3.2. Second Period (2008–2018)

A total of 410 patients were included. The patients’ characteristics are in Table 1. Five-year OS was 71% (95CI 66–76%), while 5-year EFS was 62% (95CI 57–68%).

In univariable analyses for OS (Table 2), age <1 or ≥10 y/o, compared with 1–9 y/o; WBC > 50 × 10^9^/L, compared with WBC ≤ 10 × 10^9^/L; BFM intermediate and high-risk groups; NCI high-risk groups; and poor prednisone response and poor-risk cytogenetics (HR = 7.30, *p* = 0.0016, compared with good-risk cytogenetics) were associated with poorer OS. In the multivariable analysis (Table 3), only age <1 or ≥10 y/o (HR = 1.68, *p* = 0.0088, compared with 1–9 y/0) and BFM high-risk group (HR = 6.41, *p* = 0.01, compared with standard risk) predicted OS.

In univariable analyses for EFS (Table 4), age <1 or ≥10 y/o, compared with 1–9 y/o; male sex (HR = 1.84, *p* = 0.0079, compared with females); WBC > 50 × 10^9^/L, compared with WBC ≤ 10 × 10^9^/L; BFM high-risk group, compared with standard risk; NCI high-risk groups; and poor prednisone response were associated with inferior EFS. In the multivariable analysis (Table 3), age <1 or ≥10 y/o (HR = 1.63, *p* = 0.007, compared with 1–9 y/o) and BFM high-risk group (HR = 3.48, *p* = 0.016, compared with standard risk) predicted EFS.

In multivariable analyses, patients treated in the 2008–2018 period had a higher OS (HR = 0.71, *p* = 0.011) and EFS (HR = 0.62, *p* < 0.01).

### 3.3. Time-Dependent Variables

D15 assessment was independently associated with EFS (HR = 1.41, *p* = 0.036, for M2; and HR = 1.46, *p* = 0.029, for M3, compared with M1 bone marrow). Both D33 (HR = 1.67, *p* = 0.01; 1% cutoff) and week 12 (HR = 1.92, *p* < 0.001; 0.1% cutoff) positive assessments independently predicted EFS.

There was a trend towards inferior EFS in patients who underwent cranial (HR = 1.37, *p* = 0.07), although relapse was significantly higher (HR = 1.83, *p* = 0.0017) in both multivariable analyses.

The protocol quality indicators are detailed in Table 5. Of note, induction death rates were 3.1% for the 1997–2007 period and 1.4% for the 2008–2018 period. Death in first complete remission was 12.1% in the first period (1997–2007) and 6.1% in the second one (2008–2018).

## 4. Discussion

Our study shows that adapted BFM protocols, originally designed and implemented in high-income countries, can also achieve excellent outcomes in developing countries, even though the frequency of high-risk patients was higher than previously reported. We also validate previously reported risk factors in a Brazilian socioeconomically vulnerable sample.

In the analysis of our study’s last period (2008–2018), the 5 y EFSs were 84%, 63%, and 57% for the BFM-defined standard, intermediate, and high-risk groups, respectively, which is far better than we would expect given the socioeconomic profile of our sample. For comparison, the most recent BFM-ALL-09 trial [8] has reported 90%, 76%, and 56% 5 y EFSs for the same. Most of the discrepancies can be seen in the intermediate group, where our results were poorer, with an absolute difference of 13%. Here, some of the adaptations, reducing the methotrexate dose from 5 g/m^2^ to 2 g/m^2^ and substituting conventional asparaginase for peg-asparaginase, which happened in the former because in vivo methotrexate dosing was seldom available and, in the latter, because the drug was only recently approved in Brazil, might have taken its toll. The impact of a socioeconomic deprivileged population cannot be underestimated. Also, given the unexpectedly good results in the high-risk group, comparable to the BFM, we cannot rule out some sort of misclassification (affecting the intermediate- and high-risk groups) or survival bias in the high-risk group, in which some high-risk patients with more aggressive diseases die before being diagnosed. Despite that, our results have improved from the 1997–2007 to the 2008–2018 periods, especially in the BFM high-risk group, reflecting either better supportive care in the participating hospitals or the implementation of more recent and adapted BFM protocols.

In our study, either between 1997–2007 or 2008–2018, the BFM high-risk group accounted for more than half of the patients, obviously leading to poorer results. This is surprising since, in the BFM-ALL-09 publication [8], less than 25% were high-risk patients. Even though some weak evidence points to genetic differences between Latin American and other populations [9,10,11] and that racial background can impact ALL risk classification [12], Rio de Janeiro is considered a highly miscegenated city, we actually believe that delayed diagnoses or an overestimated D8 blast count can have played a major role. Indeed, the Brazilian public healthcare structure is known to be suboptimal and inefficient [13]. We must underscore that the patients treated in these four hospitals in Rio de Janeiro have a very unfavorable socioeconomic profile, and the impact of low levels of education of patients and caregivers in ALL outcomes has already been reported [9,10,11,14]. Regardless, our induction and CR1 mortality, overall survival, and event-free survival are very close to those reported by the BFM when analyzed within the protocol risk groups.

We validated most of the classical ALL independent prognostic factors [15], like age, sex, and BFM risk group, as well as the lack of independent prognosis of T-ALL. In the landmark analysis, cranial radiotherapy, which was usually indicated in patients with CNS+ or at high risk of CNS relapse, was associated with higher relapse rates. Since cranial radiotherapy is scheduled for 6 months after diagnosis, methods like landmark analysis are needed to not incur immortal-time bias [16]. Although most modern protocols tend to avoid cranial radiotherapy, its omission should be carefully implemented in our population, particularly if adaptations of the original BFM protocols are expected to be used.

Even though it is recommended to strictly adhere to treatment protocols, this is not always possible. In our country, peg-asparaginase was only approved in 2016 and, therefore, incorporated. Likewise, methotrexate pharmacokinetics monitoring was not available in all four centers until 2017, limiting the maximum dose in centers to 2 g/m^2^ instead of 5 g/m^2^ until then. Moreover, socioeconomic disparities and significant income inequality hinder rapid access to reference centers. However, collaborative efforts among multiple healthcare stakeholders have the potential to improve this access and speed up the implementation of more effective primary care early diagnosis strategies and regulations. Despite that, acceptable results have been achieved, suggesting that local adaptations can be implemented, given that patients are included in the rigorous assessment of the results. It should be underscored that the BFM protocols are a part of studies having centralized quality control.

This study has several limitations. This is a retrospective study. Moreover, many different BFM protocols have been used without a central review of D8 and D15 assessments and no MRD standardization at D33 and week 12. However, we estimated that this study included about 85% of all predicted pediatric ALL cases diagnosed in Rio de Janeiro state (Rio de Janeiro city is the capital of Rio de Janeiro state) during the period. Another strength of our study is that all four centers used were familiar with BFM-adapted protocols.

## 5. Conclusions

In summary, we have shown that the BFM protocols can be adapted to developing countries’ conditions, and excellent results, close to those reported by the BFM study group, can be achieved in ALL patients. Developing countries should focus on establishing local ALL networks and standardizing treatments and procedures.

## Figures and Tables

**Table 1:** Patients’ characteristics.

	Period		*p* Value
	1997–2007	2008–2018	
Total	285	410	
Sex			0.22
Female	126 (44)	162 (40)	
Male	159 (56)	248 (60)	
Age			0.43
<1 or ≥10 y/o	77 (27)	122 (30)	
1–9 y/o	208 (73)	288 (70)	
Immunophenotyping			0.19
B-ALL	238 (84)	326 (80)	
T-ALL	47 (16)	84 (20)	
ALL subtype			0.75
None	168 (89)	319 (88)	
t(1,19)	6 (3)	8 (2)	
t(4,11)	7 (4)	16 (4)	
t(9,22)	8 (4)	21 (6)	
CNS			0.03
CNS-	249 (87)	333 (81)	
CNS+	36 (13)	77 (19)	
WBC (1 × 10^9^/L)			0.65
≤10	120 (45)	171 (44)	
>10 and ≤50	81 (31)	108 (28)	
>50 and ≤100	24 (9)	35 (9)	
>100	39 (15)	71 (18)	
BFM risk group			0.8
Standard risk	26 (9)	32 (8)	
Intermediate risk	111 (40)	166 (41)	
High risk	144 (51)	208 (51)	
NCI risk group			0.07
B-ALL, standard risk	111 (39)	171 (42)	
B-ALL, high risk	127 (45)	155 (38)	
T-ALL, standard risk	16 (6)	16 (4)	
T-ALL, high risk	31 (11)	68 (17)	
Cytogenetic risk			<0.001
Good	20 (11)	87 (24)	
Intermediate	147 (78)	230 (63)	
Poor	22 (12)	47 (13)	
Prednisone response			0.03
Poor	115 (42)	136 (34)	
Good	159 (58)	267 (66)	
Cranial radiotherapy			0.75
No	148 (52)	218 (53)	
Yes	137 (48)	192 (47)	

**Table 2:** Univariate overall survival analyses.

	Category	1997–2007 Period						2008–2018 Period					
		HR	95%CI	*p* (Wald)	5y-OS	95%CI	*p* (Logrank)	HR	95%CI	*p* (Wald)	5y-OS	95%CI	*p* (Logrank)
Sex	Female	1.00			69%	61–78	0.0054	1.00			73%	66–81	0.22
	Male	1.68	1.16–2.44	0.006	56%	48–64		1.28	0.86–1.90	0.22	69%	62–76	
Age	1–9 y/o	1.00			68%	61–74	<0.001	1.00			77%	71–83	<0.001
	<1 or ≥10 y/o	1.88	1.30–2.72	<0.001	45%	34–58		2.04	1.40–2.98	<0.001	56%	46–67	
Immunophenotyping	B-ALL	1.00			63%	57–70	0.18	1.00			71%	65–77	0.27
	T-ALL	1.35	0.87–2.09	0.18	53%	40–69		1.27	0.83–1.97	0.27	69%	60–80	
ALL subtype	None	1.00			68%	62–76	<0.001	1.00			72%	66–78	0.14
	t(1;19)	1.83	0.67–5.03	0.24	50%	22–100		1.88	0.69–5.13	0.22	47%	21–100	
	t(4;11)	1.86	0.68–5.10	0.23	43%	18–100		2.16	1.00–4.68	0.051	52%	30–88	
	t(9;22)	4.09	1.94–8.60	<0.001	12%	2–78		0.90	0.36–2.21	0.81	69%	49–97	
CNS	CNS-	1.00			63%	57–69	0.27	1.00			71%	66–77	0.72
	CNS+	1.32	0.81–2.15	0.27	51%	36–71		1.09	0.68–1.75	0.72	66%	55–79	
WBC (1E9/L)	≤10	1.00			68%	59–77	<0.001	1.00			79%	72–87	0.019
	>10 and ≤50	0.81	0.50–1.31	0.39	71%	62–82		1.62	0.99–2.66	0.055	68%	59–78	
	>50 and ≤100	1.76	0.95–3.25	0.071	50%	34–75		2.46	1.29–4.71	0.0064	50%	32–77	
	>100	2.44	1.52–3.91	<0.001	33%	21–52		1.87	1.09–3.20	0.023	67%	56–80	
BFM risk group	Standard risk	1.00			88%	76–100	<0.001	1.00			93%	83–100	<0.001
	Intermediate risk	2.13	0.76–6.02	0.15	75%	67–84		4.24	1.02–17.68	0.047	75%	68–84	
	High risk	5.61	2.06–15.3	<0.001	48%	40–57		7.64	1.87–31.18	0.0046	64%	57–72	
NCI_risk group	B-ALL, standard risk	1.00			78%	71–86	<0.001	1.00			81%	74–89	0.0027
	B-ALL, high risk	2.21	1.46–3.36	<0.001	51%	42–60		2.30	1.46–3.62	<0.001	60%	51–69	
	T-ALL, standard risk	1.53	0.67–3.45	0.31	62%	43–91		1.81	0.70–4.70	0.22	73%	54–100	
	T-ALL, high risk	2.55	1.43–4.54	0.0014	48%	33–69		2.10	1.20–3.67	0.0094	68%	58–81	
Cytogenetic risk	Good	1.00			95%	86–100	<0.001	1.00			75%	66–87	0.26
	Intermediate	3.50	1.10–11.2	0.034	65%	57–73		1.33	0.81–2.18	0.26	70%	64–77	
	Poor	7.30	2.12–25.1	0.0016	36%	21–63		1.70	0.89–3.27	0.11	59%	44–78	
Prednisone response	Good	1.00			74%	67–81	<0.001	1.00			78%	72–84	<0.001
	Poor	2.62	1.82–3.78	<0.001	45%	37–55		2.80	1.71–3.67	<0.001	58%	49–67	
Cranial radiotherapy	No	1.00			60%	52–69	0.89	1.00			68%	61–75	0.094
	Yes	0.98	0.69–1.38	0.89	64%	56–72		0.73	0.50–1.06	0.096	74%	67–81	

HR, hazard ratio; 95%CI, 95% confidence interval; OS, overall survival; ALL, acute lymphoblastic leukemia; CNS, central nervous system; WBC, white blood counts; BFM, Berlin–Frankfurt–Munster; NCI, National Cancer Institute.

**Table 3:** Multivariable analyses for overall and event-free survival.

Variable	Category	OS, 1997–2007			EFS, 1997–2007			OS, 2008–2018			EFS, 2008–2018		
		HR	95%CI	*p*	HR	95%CI	*p*	HR	95%CI	*p*	HR	95%CI	*p*
Age	1–9 y/o	1.00			1.00			1.00			1.00		
	<1 or ≥10 y/o	1.60	1.1–2.32	0.015	1.57	1.04–2.39	0.033	1.68	1.14–2.48	0.0088	1.63	1.14–2.33	0.007
Sex	Female	1.00			1.00			-			1.00		
	Male	1.56	1.07–2.27	0.021	1.79	1.14–2.80	0.012	-			1.28	0.89–1.84	0.18
BFM risk group	Standard risk	1.00			1.00			1.00			1.00		
	Intermediate	1.98	0.70–5.59	0.20	2.71	0.83–8.83	0.098	3.78	0.9–15.8	0.068	2.46	0.88–6.86	0.086
	High risk	4.77	1.74–13.1	0.0025	3.38	1.065–10.9	0.042	6.41	1.56–26.4	0.01	3.48	1.26–9.59	0.016

HR, hazard ratio; 95%CI, 95% confidence interval; OS, overall survival; EFS, event-free survival; WBC, white blood counts; BFM, Berlin–Frankfurt–Munster.

**Table 4:** Univariate event-free survival analyses.

	Category	1997–2007 Period						2008–2018 Period					
		HR	95%CI	*p* (Wald)	5y-EFS	95%CI	*p* (Logrank)	HR	95%CI	*p* (Wald)	5y-EFS	95%CI	*p* (Logrank)
Sex	Female	1.00			63%	55–72	<0.001	1.00			67%	59–76	0.082
	Male	1.84	1.31–2.59	<0.001	40%	33–49		1.38	0.96–1.97	0.084	58%	52–66	
Age	1–9 y/o	1.00			55%	48–62	0.001	1.00			67%	61–73	<0.001
	<1 or ≥10 y/o	1.76	1.25–2.48	0.0012	37%	28–50		1.90	1.35–2.69	<0.001	50%	42–61	
Immunophenotyping	B-ALL	1.00			52%	46–59	0.14	1.00			63%	57–69	0.16
	T-ALL	1.36	0.9–2.03	0.14	38%	27–55		1.33	0.89–1.97	0.16	58%	47–71	
ALL subtype	None	1.00			57%	50–65	0.0011	1.00			62%	56–69	0.31
	t(1;19)	1.5	0.55–4.11	0.43	50%	22–100		1.60	0.59–4.36	0.35	50%	25–100	
	t(4;11)	1.61	0.59–4.39	0.36	43%	18–100		1.72	0.8–3.69	0.17	52%	31–88	
	t(9;22)	4.01	1.9–8.46	<0.001	0%			1.46	0.74–2.89	0.27	45%	25–81	
CNS	CNS-	1.00			53%	47–60	0.016	1.00			63%	57–69	0.33
	CNS+	1.69	1.1–2.6	0.017	29%	17–48		1.23	0.81–1.85	0.33	57%	45–71	
WBC (1 × 10^9^/L)	≤10	1.00			57%	49–67	<0.001	1.00			71%	63–80	0.0052
	>10 and ≤50	0.98	0.65–1.5	0.94	57%	47–70		1.49	0.95–2.33	0.08	60%	51–72	
	>50 and ≤100	1.89	1.09–3.3	0.025	38%	22–63		2.53	1.42–4.52	0.0017	46%	31–69	
	>100	2.21	1.41–3.49	<0.001	28%	17–47		1.87	1.15–3.03	0.011	55%	44–70	
BFM risk group	Standard risk	1.00			80%	65–97	<0.001	1.00			84%	72–100	0.0019
	Intermediate risk	1.76	0.75–4.15	0.2	67%	58–76		2.75	0.99–7.64	0.053	63%	55–73	
	High risk	4.76	2.08–10.86	<0.001	33%	26–42		4.17	1.53–11.4	0.0053	57%	50–65	
NCI risk group	B-ALL, standard risk	1.00			66%	58–76	< 0.001	1.00			74%	66–82	<0.001
	B-ALL, high risk	2.28	1.56–3.33	<0.001	40%	32–50		2.33	1.54–3.51	<0.001	51%	43–61	
	T-ALL, standard risk	1.76	0.86–3.64	0.12	44%	25–76		1.80	0.76–4.28	0.18	63%	41–97	
	T-ALL, high risk	2.42	1.41–4.15	0.0013	35%	22–57		2.19	1.33–3.62	0.0022	56%	45–71	
Cytogenetic risk	Good	1.00			79%	63–100	0.0062	1.00			67%	56–79	0.15
	Intermediate	2.37	0.96–5.88	0.062	53%	46–62		1.27	0.81–1.99	0.29	60%	53–68	
	Poor	4.41	1.61–12.06	0.0039	32%	17–59		1.78	0.99–3.19	0.053	50%	36–70	
Prednisone response	Good	1.00			65%	58–73	<0.001	1.00			69%	62–76	<0.001
	Poor	2.56	1.83–3.57	<0.001	31%	23–41		2.31	1.64–3.27	<0.001	50%	42–60	
Cranial radiotherapy	No	1.00			56%	49–65	0.1	1.00			67%	61–74	0.77
	Yes	1.31	0.95–1.81	0.1	44%	36–53		1.05	0.75–1.48	0.77	57%	50–66	

HR, hazard ratio; 95%CI, 95% confidence interval; EFS, event-free survival; ALL, acute lymphoblastic leukemia; CNS, central nervous system; WBC, white blood counts; BFM, Berlin–Frankfurt–Munster; NCI, National Cancer Institute.

**Table 5:** Protocol quality indicators.

Outcome	N	First Period (%)	N	Second Period (%)	*p*
Death before CR (induction death)	9	3.1	10	2.4	0.575
Abandonment	12	4.0	5	1.2	0.017
Resistant disease	4	1.4	5	1.2	0.818
Death in the first CR	33	12.1	24	6.1	0.007
Death in first CR SRG	1	3.8	1	3.1	0.885
Death in first CR IRG	8	7.3	7	4.3	0.288
Death in first CR HRG	24	17.6	16	8.0	<0.001
Relapse	103	37.9	94	23.9	<0.001
Relapse SRG	5	19.2	3	9.4	0.288
Relapse IRG	32	29.1	36	22.1	0.192
Relapse HRG	66	48.5	55	27.6	<0.001
Isolated BM	78	28.7	77	19.5	0.006
Isolated CNS	7	2.4	4	1.0	0.154
Isolated Testes	7	2.4	4	1.0	0.154
Combined CNS/BM	5	1.7	6	1.5	0.839
Combine BM/Other	11	3.8	9	2.2	0.223
Other relapse	0	0	3	0.7	0.168
Secondary neoplasia	1	0.3	0	0	0.277
BMT in refractory disease	0		0		
BMT in CR1	5	1.7	17	4.1	0.081
BMT after relapse	28	9.8	27	6.5	0.120

BM, bone marrow; CR, complete remission; CNS, central nervous system; BMT, bone marrow transplantation; CR1, first complete remission; SRG, standard risk group; IRG, intermediate risk group; HRG, high-risk group.

## Data Availability

The unidentified database can be shared at a reasonable request by the corresponding author.
